# What Is the Value of the Learning Curve in Endoscopic Balloon Dilatation of the Major Papilla?

**DOI:** 10.1155/2017/6501485

**Published:** 2017-08-16

**Authors:** Eduardo Tavio-Hernandez, Enrique Vazquez-Sequeiros, Enrique Rodriguez-Santiago, Juan Angel Gonzalez-Martin, Jose Ramón Foruny-Olcina, Vicente Benita-Leon, Victor Defarges-Pons, Daniel Boixeda-Miquel, Agustin Albillos-Martínez

**Affiliations:** Endoscopy Unit, Gastroenterology and Hepatology Department, Hospital Universitario Ramón y Cajal, IRYCIS, Madrid, Spain

## Abstract

**Introduction:**

Endoscopic papillary large balloon dilatation (EPLBD) is an alternative for the treatment of common bile duct (CBD) stones. Existing evidence of factors associated with its outcomes is contradictory.

**Objective:**

To identify predictors (including the experience of an endoscopist) of success and adverse events in EPLBD.

**Methods:**

We reviewed the first 200 EPLBD with endoscopic sphincterotomy (EST) performed at our center. Demographic, clinical, and anatomic variables were studied, as well as the performance characteristics, correlating them with individual and group experience.

**Results:**

Global success was obtained in 87% of cases, and adverse events occurred in 16% of cases. Success was associated with stone size, CBD diameter, and the need to perform mechanical lithotripsy (ML). Despite that adverse events were not univariately associated with any factor, severe adverse events were more likely to occur in stones > 13.5 mm. Multivariate analysis which disclosed success was higher when ML was not required and stones were < 13.5 mm. It also showed that no factor was associated with adverse events or their severity. No differences were found on success or adverse events that could be directly related to experience.

**Conclusions:**

Success of EPLBD-EST is higher in stones < 13.5 mm and when ML is not required. Experience does not appear to play a major role.

## 1. Introduction

Treatment of common bile duct (CBD) stones is, at present time, the most frequent indication to perform endoscopic retrograde cholangiopancreatography (ERCP) in Western countries [1]. For this purpose, we have a variety of endoscopic techniques of proven efficacy. Among them, the most frequently used are endoscopic sphincterotomy (EST) and mechanical lithotripsy (ML) that facilitate stone removal with a Fogarty balloon or a Dormia basket [2].

Endoscopic papillary large balloon dilatation (EPLBD) has emerged in recent years as an alternative or complement to EST, which was classically considered the standard therapy [3]. EPLBD was initially developed under the hypothesis that it could potentially be less traumatic than EST to the major papilla, and therefore, it could be associated with fewer adverse events [3]. Initial studies showed that EPLBD was indeed associated with less bleeding but an increased risk of acute pancreatitis [4, 5].

However, EPLBD in patients with prior EST has been demonstrated in several meta-analyses [6–10], to have a safety profile and effectiveness similar to EST, and may be even more cost-effective, by reducing the number of ERCPs required to completely clean the CBD in patients with multiple or large stones [8, 11–13]. Furthermore, some recent studies have suggested that EPLBD may be equally effective even in patients with no EST [14–16]. For these reasons, EPLBD has become accepted in clinical practice as a solid alternative to EST when treating large CBD stones [2, 17], and some authors may even consider EPLBD as the first option in those patients [8, 12].

Although there are some data in the literature [18] regarding potential predictors of therapeutic success and development of adverse events after EPLDB with EST (EPLBD-EST), our knowledge in this field is still limited. Furthermore, it is unknown if the experience of the endoscopist performing the EPLBD influences the success and safety of the technique or if there is a learning curve to safely perform an EPLBD.

## 2. Objectives

The objectives of our study are to identify predictors of success and adverse events in EPLBD-EST and to determine if the experience of the endoscopist is associated with technical success or adverse events in patients undergoing EPLBD-EST for CBD stone extraction.

## 3. Materials and Methods

**Figure 1:**
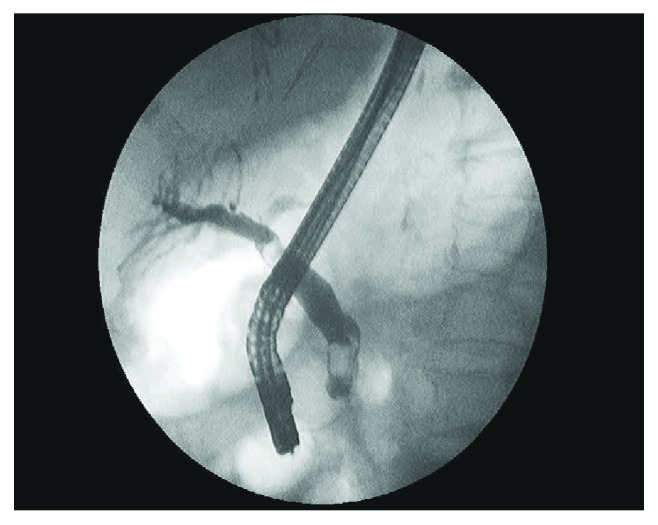
Common bile duct stones on X-ray fluoroscopy.

The study was conducted at a tertiary referral hospital. Retrospective analysis of a database including all ERCP procedures was performed at that center over an 8-year period (October 2007–May 2015). The first 200 EPLBD-EST performed at that institution (endoscopists with no previous experience on EPLBD), for the treatment of large CBD stones (defined as stones larger than the sphincterotomy that were not amenable to be removed by a Dormia basket or a Fogarty balloon) (Figure 1), were included. EST was performed prior to EPLBD in all cases, either during the same ERCP or in a previous one.

**Figure 2:**
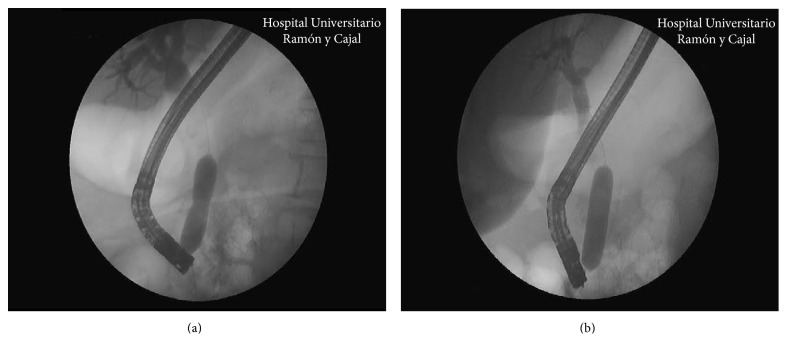
Partially (a) and totally (b) inflated balloon dilatation catheter on X-ray.

**Figure 3:**
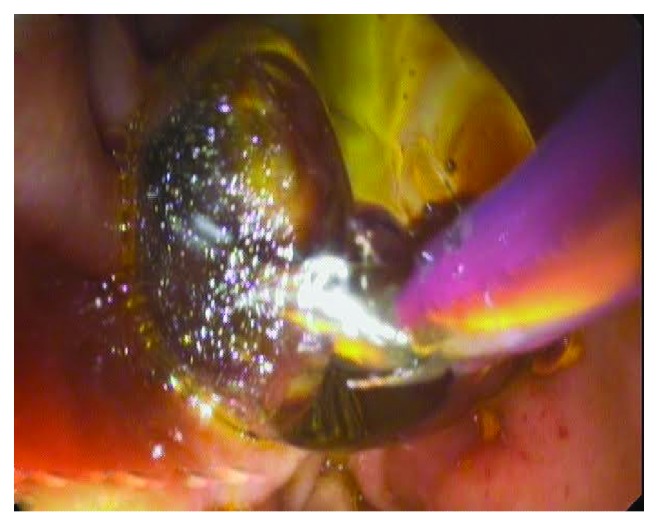
Inflated balloon dilatation catheter in direct endoscopic vision.

Duodenoscopes from Olympus® (TJF-160 VR Olympus Medical Systems, Tokyo, Japan) and Pentax® ED-3490TK (Pentax Medical, Tokyo, Japan) were employed for ERCP performance. EPLBD was performed by using a hydropneumatic balloon (CRE Balloon Dilatation Catheter, Boston Scientific®, Marlborough, United States), with diameters ranging from 10 to 20 mm (Figures 2 and 3). This balloon was centered at the major papilla, and forced dilatation was obtained for 30 to 60 seconds or until the wrist of the papilla disappeared on the X-ray image. The maximum diameter of dilatation was decided by the endoscopist based on the size of the stone and papilla.

The Hospital Ethics Committee approved this retrospective study, and informed consent for ERCP and EPLBD-EST was obtained in all cases. All procedures were performed on an inpatient basis. Clinical and laboratory data were obtained from the patient's electronic medical history. EPLBD-EST was performed by 5 different endoscopists; each of them had previously performed more than 300 ERCPs but had no experience on EPLBD at the beginning of the study. Therefore, this study represents their learning curve on EPLBD-EST. The following variables were included for the analysis: indication for ERCP, age, sex, patient's condition based on the American Society of Anaestesiology (ASA classification) [19], and laboratory parameters (renal and hepatobiliary function, blood count, and coagulation). We also collected information regarding the size and number of CBD stones, diameter of the CBD, presence of periampullary diverticulum or gastrectomy with Billroth II reconstruction, if EST was performed at the same time or previously and if precut sphincterotomy was required, maximum diameter of dilatation, need to use ML for stone removal, and degree of difficulty of ERCP according to the ASGE classification [20].

Adverse events were investigated from electronic medical history, and their severity was classified as mild, moderate, or severe according to criteria proposed by Cotton et al. [21] (Table 1), which takes into account the consequences of the adverse events and the specific care required.

To analyze the effect of experience on success and adverse events associated with EPLBD-EST, different studies and comparisons were conducted:

1. Individual success and adverse event rate of the 5 endoscopists participating in this study were compared.
2. Individual learning curve of each of the 5 endoscopists: first half (50% of their EPLBD-EST) versus second half, was compared to investigate how experience influenced each endoscopist's performance in terms of success and adverse events.
3. Overall group learning curve: the influence of experience on the entire group was investigated by analyzing all 200 cases by groups of 50 consecutive patients (group A: 0–50th patient; group B: 51–100th patient; group C: 101–150th patient; and group D: 151–200th patient).

### 3.1. Definitions Adopted in the Study

**Figure 4:**
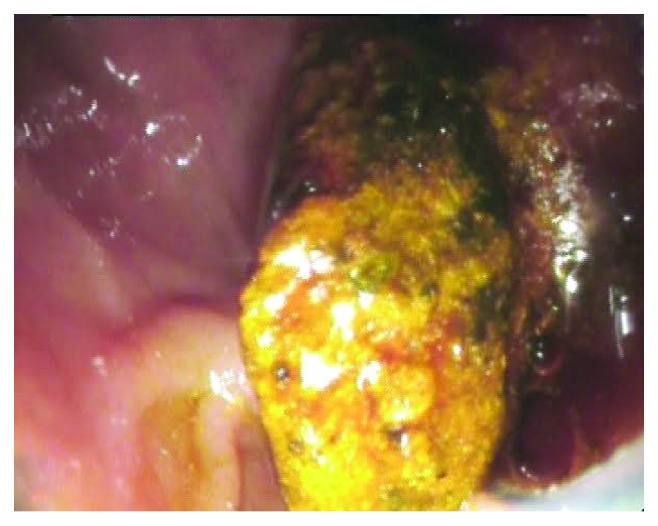
Common bile duct stone removal in direct endoscopic vision.

1. Treatment success: completion of all procedures involved in the EPLBD-EST (Figure 4), followed by a cholangiography showing no filling defect, and a clinical and analytical surveillance with no evidence of recurrence within the first 6 months.
2. Treatment failure: impossibility to clean up the CBD after EPLBD-EST completion, or analytical/clinical recurrence within the first 6 months.

### 3.2. Statistical Analysis

Quantitative variables are presented as mean and standard deviation or median and interquartile range. The qualitative variables are expressed as absolute values and percentages. Univariate analysis of quantitative data was conducted by using the Student *t*-test (parametric data) and the Mann–Whitney *U* test (nonparametric data). Discrete variables were analyzed with the chi-square test and Fischer's exact test. Multivariate analysis was performed by using multiple stepwise logistic regression analysis. Variables that were statistically significant in the univariate analysis or showed some trend towards significance were included in the multivariate analysis.

The 11.0-SAS JMP statistical software was used for calculations. A *p* value of <0.05 was considered to be statistically significant. With respect to sample size, as no data was available on the effect of experience on EPLBD-EST outcomes, we arbitrarily hypothesized that a sample size of 200 ERCP with EPLBD-EST may probably provide a sufficient statistical power to identify trends in outcomes over time that may be related to the effect of the learning curve of the technique.

## 4. Results

As previously described, we included 200 consecutive EPLBD-EST in the study period. Patient baseline characteristics, indications for EPLBD-EST, and ERCP data are displayed on Table 2.

The average size of stones, as measured by the X-ray images, in this cohort of patients was 13.5 ± 4.7 mm. In 54 of 200 patients (27%), a single stone was observed, while the remaining patients had a larger number of CBD stones. Seventy-five patients included in this cohort (37.7%) had a previous EST, while in the remaining cases, an EST was performed in the same session of the EPLBD. It was necessary to perform a precut sphincterotomy in 9 cases (4.5%) and ML in 13 cases (6.5%) to successfully complete the procedure.

The degree of difficulty of ERCP, according to of the ASGE grading system [20], was as follows: grade 1 in 2 cases (1%); grade 2 in 50 cases (25%); grade 3 in 138 cases (69%); and grade 4 in 10 cases (5%).

Overall, complete cleaning of the CBD was achieved in a single session in 165 of 200 patients (82.5%), increasing up to 174 of 200 patients (87%) if a second session of ERCP with EPLBD-EST was performed.

Adverse events occurred in 32 of 200 patients (16%): acute pancreatitis (12/200: 6%), bleeding (9/200: 4.5%), perforation (6/200: 3%), cholangitis (4/200: 2%), and a respiratory adverse event related to sedation (1/200: 0.5%). Adverse events were classified as mild (19/32: 59%), moderate (8/32: 25%), or severe (5/32: 16%) according to the Cotton classification [21].

Results of the univariate analysis of factors that could be associated with the success or development of adverse events and their severity are displayed in Tables 3(a) and 3(b). Success was univariately associated with stone size < 13.5 mm, CBD diameter < 14.1 mm, and cases where ML was not required (*p* < 0.05) (Table 3(a)). Endoscopists number 2 and number 3 were also associated with a higher probability of success (“high profilers”) as compared with the other three endoscopists (“low profilers”) (*p* < 0.05) (Table 3(b)). Adverse event developments were not univariately associated with any factors (*p* > 0.05), while severe adverse events were univariately associated with stones ≥ 13.5 mm in size (*p* < 0.05). Endoscopists identified after the study as “*high and low profilers*” for EPLBD-EST had no significant differences in age (all of them in the 4th and 5th decades) or ERCP experience (>5 years and >300 ERCP procedures before the study) (*p* > 0.05).

Multivariate analysis of success, adverse events, and severity of them (Table 3(c)) showed that stones < 13.5 mm in diameter and cases where ML had not been required were both independently associated with EPLBD-EST success. Adverse events and the severity of them were not associated in the multivariate analysis with any factor.

Specific analysis conducted to investigate how an endoscopist's experience influenced EPLBD-EST success showed that although endoscopists number 2 (93.8%) and number 3 (90%) were compared favorably with endoscopists number 1 (71.4%), number 4 (79.5%), and number 5 (70%) (*p* = 0.03) (Table 3(b)), none of the five endoscopists significantly improved their success rate in the second part of their personal learning curve (Table 4(a)). Furthermore, when the experience of the group of 5 endoscopists was globally analyzed, a significant improvement in success could not be demonstrated over time (*p* = 0.33) (Table 4(b)). Same negative results were found when adverse events were analyzed by endoscopists (*p* = 0.9) (Table 3(b)), individual learning curve of each endoscopist (*p* > 0.05) (Table 4(a)) and overall group learning curve (*p* = 0.44) (Table 4(b)).

## 5. Discussion

Results of the present study, with an 82.5% efficacy rate in the first session, 87% in the second EPLBD-EST, and an adverse event rate of 16%, are similar to previous studies published in the literature [11, 15, 22–26]. Results support EPLBD-EST as an efficient and relatively safe technique for those patients with difficult stones. Results from the present retrospective cohort study suggest, for the first time in the literature, that experience in EPLBD-EST may play a minor role.

As expected, large stones (≥13.5 mm in size) were more difficult to remove from the CBD, therefore requiring more frequently ancillary maneuvers like ML to successfully complete EPLBD. Other factors being analyzed, like periampullary diverticulum or Billroth II reconstruction which have been reported to decrease procedural success in other series [24, 27–29], did not appear to be associated with EPLBD success. Therefore, in our opinion, the presence of any of these anatomical variants should not discourage the endoscopist to consider performing this useful technique. In this retrospective cohort study, none of the factors investigated was significantly associated with adverse events after EPLBD-EST nor was associated with the severity of them. Whether these negative results may be due to a limited statistical power or reduced sample size cannot be assessed by this study, but it is a possibility that cannot be completely excluded [18]. Furthermore, potential bias introduced by the retrospective nature of the study is also a possibility that we can neither be sure nor modify but should certainly be considered. Results of the present study, consistent with prior literature [15, 30], suggest that the clinical status of the patient (age, ASA grade, and comorbidities) is not related with adverse events and should not influence the decision of EPLBD-EST performance.

Regarding experience on EPLBD-EST and its learning curve, this study provides us with novel and unknown data that we believe may be of interest in this unexplored topic. As 5 different endoscopists started performing EPLBD-EST at the same time, we have been able to document how they performed over time. Results of the present study support, in our opinion, the following statements: (1) success of EPLBD-EST, but not adverse events, may depend on the endoscopist performing it (Table 3(b)); (2) the success and adverse event rate of each of the endoscopists do not improve as experience is gained (first half vesus second half of their EPLBD-EST learning curve) (Table 4(b)); and (3) the success and adverse event rate of the whole group do not improve over time as experience is gained (Table 4(b)).

To our knowledge, these three points had never been proven before and may certainly provide important information for clinical practice. First of all, endoscopists are not equally effective when performing EPLBD-EST. This may be due to varying technical skills or endoscopist “aggressiveness” (determination of success on bile duct stone clearance), which is, in our opinion, difficult to modify. This study demonstrates that, contrarily to the learning curve of ERCP [31, 32], the efficacy and safety of EPLBD-EST does not appear to be influenced by the experience of the endoscopist. One may even argue that these data support the hypothesis that the learning curve to perform EPLBD-EST is probably not clinically relevant in experienced ERCP endoscopists. Moreover, in our opinion, the lack of experience in EPLBD-EST should not discourage endoscopists to perform it, because its profile of success and complications is not related to experience. It is possible that these findings may have been influenced by a high baseline experience in ERCP. Whether or not these conclusions may be applicable to ERCP beginners is beyond the scope of the present study.

When designing the present study, we had hypothesized that with increasing experience, one may gain confidence at selecting the appropriate size of the balloon for EPLBD-EST and this could finally result in a more efficient and safe intervention. However, this was not found to be the case in this retrospective cohort study. In our opinion, this novel information is of particular value. As shown in Table 4(a), “high profilers” were equally effective at the beginning and at the end of their learning curve (endoscopist number 2: 86% versus 100% and endoscopist number 3: 91% versus 89%). “Low profilers” in general did not show improvement in the second half (endoscopist number 1: 71% versus 71%, endoscopist number 4 80 versus 79%, and endoscopist number 5 60% versus 88%). In other words, outcomes appear to be related to specific endoscopist profile and their results will not improve as experience is gained.

We have to acknowledge that the present study may be limited by the retrospective nature of its design. This may have introduced selection or recall bias that may have influenced results. On the other hand, we would to like point out that this study may represent the extended experience of a group of experienced endoscopists and describe from its beginning the evolution of EPLBD-EST at their institution. Our study, as previously mentioned, may also be limited by a limited statistical power to detect differences in adverse events due to their relatively low incidence. This may have somehow underestimated, in our opinion, the influence of experience on adverse events, but it is less likely when evaluating success.

In summary, EPLBD-EST is a technique of proven efficacy for the treatment of large CBD stones. The size of the stone appears to be the most important factor to predict success when performing EPLBD-EST. The endoscopist's experience does not appear to play a relevant role in the therapeutic success and adverse event rate of this technique. Results from the present study suggest that in the case of experienced endoscopists, the learning curve of EPLBD-EST may have little importance. Larger prospective and controlled studies are required to definitively solve this question.

## Figures and Tables

**Table 1:** Grading system for major complications of ERCP according to Cotton et al. [21].

	Mild	Moderate	Severe
Bleeding	Hemoglobin drop <3 g and no need for transfusion	Transfusion (4 units or less), no angiographic intervention or surgery	Transfusion (5 units or more) or intervention (angiographic or surgical)
Perforation	Possible or only very slight leak of fluid or contrast, treatable by fluids and suction for 3 days or less	Any definite perforation treated medically for 4–10 days	Medical treatment for more than 10 days or intervention (percutaneous or surgical)
Pancreatitis	Requires admission or prolongation of planned admission to 2-3 days	Pancreatitis requiring hospitalization for 4–10 days	Hospitalization for more than 10 days, local complication or intervention (percutaneous drainage or surgery)
Cholangitis	>38°C, 24–48 hr	Febrile or septic illness requiring more than 3 days of hospital treatment or endoscopic or percutaneous intervention	Septic shock or surgery

**Table 2:** Patient baseline characteristics and ERCP findings.

Sex: F (%)/M (%)	102 (51%)/98 (49%)				
Age (years)	74.3 ± 14.8 [21–97]				
Platelet count (mcl)	241460 ± 90000 [54000–638000]				
Bilirubin (mg/dl)	3.05 ± 4.1 [0.32–24.6]				
Alkaline phosphatase (UI/ml)	308 ± 258 [43–1602]				
GGT (UI/ml)	517 ± 430 [14–2018]				
INR	1.03 ± 0.15 [0.8–1.8]				

ERCP indication, *n* (%)	Cholangitis, 67/200 (33.5%)	Jaundice, 21/200 (11.5%)	Pain, 19/200 (9.5%)	Pancreatitis, 12/200 (6%)	Cholangitis-pancreatitis, 4/200 (2%)

ASA classification, *n* (%)	ASA 1, 24/200 (12%)	ASA 2, 70/200 (35%)	ASA 3, 82/200 (41%)	ASA 4, 24/200 (12%)	

Number of stones, *n* (%)	1 stone, 71/200 (35.5%)	2 stones, 32/200 (16%)	3–5 stones, 38/200 (19%)	6–10 stones, 34/200 (17%)	>10 stones, 25/200 (12.5%)

Stone size (mm)	13.5 ± 4.7 [5–40]				
CBD size (mm)	14.1 ± 5.9 [6–40]				

ERCP difficulty, *n* (%)	Grade 1, 0/200 (0%)	Grade 2, 52/200 (26%)	Grade 3, 138/200 (69%)	Grade 4, 10/200 (5%)	

PAD, *n* (%)	34/200 (17%)				
Billroth II gastrectomy, *n* (%)	8/200 (4%)				
Previous EST, *n* (%)	75/200 (37.5%)				
Need for precut EST, *n* (%)	9/200 (4.5%)				
Need for ML, *n* (%)	13/200 (6.5%)				
Diameter of dilatation (mm)	14.1 ± 2 [8–19]				

F: female; M: male; INR: international normalized ratio; GGT: gamma glutamil transpeptidase; ASA: American Society of Anaesthesiologists; CBD: common bile duct; PAD: periampullar diverticulum; EST: endoscopical sphincterotomy; EPLBD: endoscopic papillary large balloon dilatation; ML: mechanical lithotripsy.

**Table:** (a) Factors associated with success, adverse events, and severity. Univariate analysis

	Success (%)	*p* value	Adverse events (%)	*p* value	Proportion of severe adverse events (%)	*p* value
Age (<74.3 versus ≥74.3)	78.3/84.7	0.32	15.9/16	0.9	9.1/19	0.07
Sex (male/female)	84.7/80.4	0.57	15.3/16.7	0.81	20/11.8	0.72
INR (<1.03 versus ≥1.03)	81.3/85.5	0.8	15.8/14.5	0.34	13.6/25	0.49
Bilirubin (<3.05 versus ≥3.05)	83.1/79.2	0.13	16.2/15.1	0.94	21.7/0	0.99
Alkaline phosphatase (<307 versus ≥307)	79.6/86.6	0.62	11.1/23.9	0.08	8.3/18.8	0.3
GGT (<517 versus ≥517)	84.9/79.1	0.13	11.3/23.9	0.11	16.7/12.5	0.12
ASA classification (1/2/3/4)	66.7/85.9/85.2/79.2	0.18	20.8/15.5/13.6/20.8	0.75	0/18.2/27.3/0	0.3
PAD (yes/no)	85.3/81.9	0.61	14.7/16.3	0.85	0/18.5	0.48
Billroth-II gastrectomy (yes/no)	87.5/82.3	0.69	37.5/15.1	0.08	33.3/13.8	0.56
ERCP indication (cholangitis versus others)	88.1/79.7	0.56	11.9/18	0.51	40/4.5	0.12
≥2 stones (yes/no)	81.7/82.9	0.43	16.9/15.5	0.48	33.3/5	0.09
Size of stones (<13.5 versus ≥13.5 mm)	91.5/71.9	<0.001	17/14.6	0.51	6.2/23.1	0.04
CBD diameter (<14.1 versus ≥14.1 mm)	91.2/72.6	<0.001	13.2/16.4	0.4	16.7/8.3	0.63
Dilation diameter (<14.1 versus ≥14.1 mm)	88.5/78.7	0.07	12.8/18	0.34	0/22.7	0.19
EST (same time) (yes/no)	80.3/88.5	0.3	16.3/15.4	0.86	8.3/37.5	0.05
Previous EST (yes/no)	89.3/79	0.1	14.7/16.9	0.67	27.3/9.5	0.2
Previous EPLBD (yes/no)	83.3/82.4	0.92	16.7/16	0.92	0/16.7	0.48
Need for ML (yes/no)	46.2/85	<0.001	7.7/16.6	0.4	0/16.1	0.7
Precut EST (yes/no)	77.8/82.7	0.71	22.2/15.7	0.58	50/13.3	0.33
ERCP difficulty (1/2/3/4)	100/92/79/80	0.19	0/16/15.9/2	0.95	0/0/18.2/5	0.18

INR: international normalized ratio; GGT: gamma glutamil transpeptidase; ASA: American Society of Anaesthesiologists; CBD: common bile duct; EST: endoscopical sphincterotomy; EPLBD: endoscopic papillary large balloon dilatation; ML: mechanical lithotripsy; PAD: periampullary diverticulum.

**Table:** (b) Outcomes depending on each endoscopist

	Total	Endoscopist number 1	Endoscopist number 2	Endoscopist number 3	Endoscopist number 4	Endoscopist number 5	*p* value
Number of EPLBD	200	42	29	70	39	20	
Success (EPLBD 1st session)	165/200 (82.5%)	30/42 (71.4%)	27/29 (93.1%)	63/70 (90%)	31/39 (79.5%)	14/20 (70%)	*0.03*
Adverse events (EPLBD 1st session)	32/200 (16%)	7/42 (16.7%)	6/29 (20.7%)	11/70 (15.7%)	6/39 (15.4%)	2/20 (10%)	0.9

EPLBD: endoscopic papillary large balloon dilatation.

**Table:** (c) Factors associated with technical success, complications, and their severity. Multivariate analysis

	OR	95% CI	*p*
*Technical success*			
Need for ML	0.19	0.04–0.81	*0.03*
Size of stones (<13.5/≥13.5 mm)	4.31	1.49–14.2	*0.01*
CBD diameter (<14.1/≥14.1 mm)	1.98	0.76–5.35	0.17
Dilation diameter (<14.1/≥14.1 mm)	0.94	0.27–3.34	0.93
Endoscopists 2 and 3	0.48	0.17–1.36	0.16
*Incidence of adverse events*			
Endoscopists 2 and 3	0.99	0.30–2.80	0.91
Billroth-II gastrectomy	2.14	0.39–10.13	0.34
Platelet count	0.39	0.15–0.94	*0.04*
Alkaline phosphatase	0.48	0.20–1.13	0.09
*Severe adverse events*			
Age > 74.3	0.9	0.008–82.9	0.96
>2 stones	*0.07*	0.001–1.03	*0.09*
Size of stones (<13.5/≥13.5 mm)	9.46	0.26–4224.83	0.31
EST (same time)	8.64	0.41–450.42	0.18

ML: mechanical lithotripsy; CBD: common bile duct; GGT: gamma glutamil transpeptidase; EST: endoscopical sphincterotomy.

**Table:** (a) Individual learning curve: success and adverse events rate of each individual endoscopist. First and the second halves of their EPLBD learning curve compared

	Endoscopist number 1		Endoscopist number 2		Endoscopist number 3		Endoscopist number 4		Endoscopist number 5		*p* value
	*1st half*	*2nd half*	*1st half*	*2nd half*	*1st half*	*2nd half*	*1st half*	*2nd half*	*1st half*	*2nd half*	
Success rate, *n* (%)	15/21 (71%)	15/21 (71%)	13/15 (86%)	14/14 (100%)	32/35 (91%)	31/35 (89%)	16/20 (80%)	15/19 (79%)	6/10 (60%)	8/10 (80%)	*p* > 0.05
Adverse event, *n* (%)	2/21 (10%)	5/21 (24%)	3/15 (21%)	3/14 (20%)	6/35 (17%)	5/35 (14%)	3/20 (15%)	3/19 (16%)	0/10 (0%)	2/10 (20%)	*p* > 0.05

**Table:** (b) Overall group learning curve: success and adverse events rate of the entire group over time. Analysis was conducted by comparing groups of 50 consecutive patients

	0–50 cases	51–100 cases	101–150 cases	151–200 cases	*p* value
Success rate, *n* (%)	39/50 (78%)	35/50 (70%)	39/50 (78%)	40/50 (90%)	0.33
Adverse event rate, *n* (%)	5/50 (10%)	10/50 (20%)	10/50 (20%)	7/50 (14%)	0.44

## Abbreviations

ERCP: Endoscopic retrograde cholangiopancreatography
EPLBD: Endoscopic papillary large balloon dilatation
CBD: Common bile duct
EST: Endoscopic ephincterotomy
ML: Mechanical lithotripsy.
